# Improved organ absorbed dose estimation in abdominal CT using DICOM header‐based tube current modulation profiles: Validation with measurements and Monte Carlo simulations

**DOI:** 10.1002/acm2.70321

**Published:** 2025-10-31

**Authors:** Chatnapa Nuntue, Kosuke Matsubara, Shu Watanabe, Kotaro Fukushima, Khajonsak Tantiwetchayanon

**Affiliations:** ^1^ Department of Quantum Medical Technology, Division of Health Sciences Graduate School of Medical Sciences Kanazawa University Kanazawa Ishikawa Japan; ^2^ Department of Radiological Technology Faculty of Allied Health Sciences Thammasat University Pathumthani Thailand; ^3^ Department of Quantum Medical Technology Faculty of Health Sciences Institute of Medical Pharmaceutical and Health Sciences Kanazawa University Kanazawa Ishikawa Japan; ^4^ Division of Nuclear Medicine Department of Radiology Faculty of Medicine Khon Kaen University Khon Kaen Thailand; ^5^ Present address: Department of Radiology Itoigawa General Hospital Itoigawa Niigata Japan; ^6^ Present address: Department of Radiological Technology Toyota Kosei Hospital Toyota Aichi Japan

**Keywords:** Computed tomography, Monte Carlo simulation, organ dose, photoluminescent glass dosimeter, voxel phantom

## Abstract

**Purpose:**

Precise determination of organ absorbed doses in computed tomography (CT) is vital for evaluating radiation risk and optimizing radiation dose. This study aimed to improve the accuracy of organ absorbed dose estimates in abdominal CT by integrating tube current modulation (TCM) profiles—extracted from the Digital Imaging and Communications in Medicine (DICOM) headers—into Monte Carlo (MC) simulations. The proposed study was validated by comparing the simulation results with direct measurements.

**Methods:**

The Particle and Heavy Ion Transport code System (PHITS), an MC simulation model, was validated by comparing its simulated weighted CT dose index (CTDI_w_) with measurements obtained using a 32‐cm PMMA phantom on a 256‐slice multidetector CT scanner. Organ doses were measured using radiophotoluminescence glass dosimeters placed at the anatomical locations of various organs within an abdominal phantom. Scans were performed under both fixed tube current conditions and TCM with noise indices (NI) of 9 and 11. A voxel‐based phantom model was constructed from CT image data, and TCM profiles were extracted from DICOM headers for use in the simulations.

**Results:**

The simulated CTDI_w_ values were within 10% of the measured data. The relative differences between simulated and measured organ absorbed doses for the liver, gallbladder, stomach, spleen, kidneys, and pancreas ranged from −14.0% to 7.87% across both fixed tube current and TCM protocols. Slightly larger deviations were noted for the kidneys under TCM at NI 9. While the average dose differed depending on the NI setting, the dose distribution trend under TCM was comparable to that of fixed tube current scans.

**Conclusion:**

The agreement between simulated and measured organ doses, including CTDI_w_, was consistent across all protocols. The observed consistency in dose distribution patterns affirms the reliability and applicability of the proposed correction method for accurate and protocol‐specific organ dose estimation in abdominal CT.

## 1 INTRODUCTION

Computed tomography (CT) is currently one of the most widely used techniques in medical imaging.^1^ As reported in Volume 1 of the UNSCEAR 2020/2021 Report, CT scans constituted roughly 10% of all imaging procedures but were responsible for 61.6% of the collective effective dose.^2^ While CT provides significant diagnostic benefits, it also exposes patients to a higher radiation dose than conventional radiography due to multiple projection angles.^3^ The effective dose from CT can range between 2 and 16 mSv, depending on the body region being scanned.^4^ Among these, abdominal CT is notable for delivering a relatively high effective dose compared to other diagnostic CT examinations, such as head or chest CT, and for involving several radiosensitive organs.^5^ Therefore, it is essential to optimize the patient's radiation dose.

Tube current modulation (TCM) is a widely adopted method for reducing patient radiation dose. TCM adjusts the tube current throughout the CT scan according to patient‐specific factors such as body size, shape, and x‐ray attenuation, while preserving diagnostic image quality. TCM generally uses three types of modulation strategies: angular modulation (in the x‐y plane), longitudinal modulation (along the z‐axis), and a combination of both.^6^ Estimating radiation dose when using TCM is complex due to the nature of the modulation, particularly when both angular and longitudinal modulation are applied simultaneously. Furthermore, the implementation of these algorithms varies significantly across CT system manufacturers.^7^

The volumetric CT dose index (CTDI_vol_) is a commonly used metric for assessing radiation dose in CT imaging. However, its applicability is limited by its physical design, as it is based on two cylindrical polymethyl methacrylate (PMMA) phantoms that simulate the human head and body without reflecting individual patient size or anatomical variations.^8^ In recent years, organ absorbed dose has increasingly been used as an alternative to effective dose for evaluating radiation risk to specific organs.^4^ Therefore, improving the accuracy of dose quantification is essential for ensuring patient safety.^9^

Several approaches have been employed to estimate organ absorbed doses, including direct measurement using small dosimeters in phantoms,^10^ such as thermoluminescent dosimeters (TLDs) and radiophotoluminescence glass dosimeters (RPLDs). RPLDs offer several advantages due to their high sensitivity, good reproducibility, minimal signal fading, and the ability to be read multiple times before annealing.^11^ These characteristics provide benefits over TLDs. Another widely used technique is the Monte Carlo (MC) simulation method, which enables accurate and reliable estimation of patient dose during CT examinations.^12^ In MC simulations, models of the CT scanner and x‐ray source are developed and must be validated against physical measurements to ensure accuracy.^13^ Previous studies have compared absorbed dose measurements with MC simulation results—with and without the TCM^9^, ^14^, ^15^, ^16^— using various MC software platforms, including GEANT4, ImPACT, NCICT (National Cancer Institute dosimetry system for Computed Tomography), and MCNPX (Monte Carlo N‐Particle eXtended code). Nevertheless, there are certain limitations inherent to simulation software, including differences in phantom modeling and variability in user interface platforms. Additionally, MC simulation models for CT that include TCM require accurate TCM profile data. Angel et al.^17^ estimated organ absorbed dose by extracting TCM profiles from the raw projection data of clinical cases and incorporating them into the simulation through photon weight adjustments based on the TCM values. Matsubara et al.^18^ carried out MC dose calculations that reflected both angular and longitudinal TCM, using profiles obtained with assistance from the scanner manufacturer. However, extracting tube current data from raw projections or log files poses challenges for users, as it typically requires access to system logs and technical support from vendors. Moreover, Israel et al.^19^ employed the ImPACT MC simulation to estimate organ absorbed doses in 91 patients who underwent CT with TCM, using tube current values extracted from the image data. However, the difference between the estimated and actual absorbed doses was not known and could not be assessed in that study.

The Particle and Heavy Ion Transport code System (PHITS), an MC simulation code, is capable of simulating all types of ionizing radiation—including photons, electrons, protons, neutrons, and heavy ions—across a broad range of energies.^20^ Moreover, it offers users a high degree of flexibility in defining model geometries, which enables the integration of anatomical models for detailed, patient‐specific dosimetry simulations. Although PHITS has been utilized for estimating organ absorbed doses in CT examinations, only a limited number of studies have incorporated TCM profiles extracted from the Digital Imaging and Communications in Medicine (DICOM) header. These profiles offer a readily accessible source of tube current data and were used in simulations as weighting factors to modulate the contribution of simulated photons for each projection angle. This approach presents a practical alternative for dose estimation and supports the advancement of patient‐specific dosimetry in CT imaging. Accordingly, the aim of this study was to improve the accuracy of organ absorbed dose estimation in abdominal CT with TCM by incorporating tube current profiles derived from the DICOM header into MC simulations and to evaluate the method's validity by comparing the results with direct measurements.

## 2 MATERIALS AND METHODS

### 2.1 Anthropomorphic phantom

**FIGURE 1:**
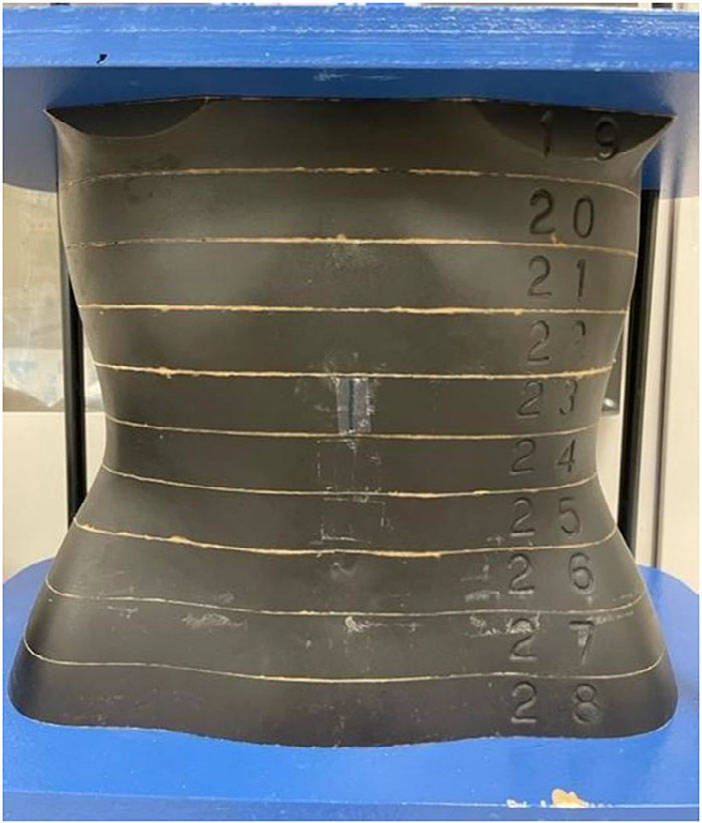
Abdominal anthropomorphic phantom.

The abdominal region of the Alderson RANDO female phantom (RAN‐110, The Phantom Laboratory, Salem, NY, USA) was utilized to replicate the anatomical features of an adult female abdomen. This phantom includes components simulating natural human bone, lung, and soft tissues and represents a female body with a height of 163 cm and a weight of 54 kg. It consists of axial slices at 25 mm intervals (Figure 1). Each slice contains a predrilled grid, allowing for the placement of small cylindrical dosimeters, such as RPLDs.

### 2.2 RPLDs and correction factor

**FIGURE 2:**
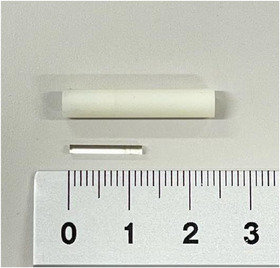
Radiophotoluminescence glass dosimeter (RPLD) with custom holder.

RPLDs were employed to measure organ absorbed doses (AGC Techno Glass, Shizuoka, Japan). Each RPLD consisted of a silver‐activated phosphate glass rod (AgPo), with dimensions of 1.5 mm in diameter and 12 mm in length. These were inserted into custom holders measuring 5 mm in diameter and 25 mm in length, which were designed to fit the holes in the phantom (Figure 2). Prior to irradiation, the RPLDs were annealed at 400°C for 30 min. After exposure, they were preheated at 70°C for 30 min to stabilize the luminescent centers. The dose readout was performed within 24 h post‐irradiation using an FDG‐1000 reader (AGC Techno Glass, Shizuoka, Japan).

The RPLDs were calibrated using an ionization chamber dosimeter (10 × 6‐0.6CT; Radcal Corporation) with an active volume of 0.6 cm^3^, connected to an analyzer (ACGM+; Radcal Corporation, Monrovia, CA, USA). The ionization chamber was placed at the isocenter of the CT scanner and irradiated twice—once before and once after the RPLDs were exposed. During irradiation, the RPLDs were mounted on a suspended plastic tape aligned with the CT gantry, ensuring their in‐air positioning was consistent with that of the ionization chamber. The CT scan parameters used for calibration were 120 kVp tube voltage, 100 mA tube current, 1 s/rot, and 80 mm beam width, all performed in non‐helical mode. The average of five readings from each RPLD was used to calculate its correction factor (*C*) using the following equation:

(1) $$C=\frac{K_{ic}}{K_{RPLD}}$$

where *K_ic_* represents the average air kerma measured by the ionization chamber (mGy), and *K*_RPLD_ is the average air kerma obtained from each RPLD (mGy).

### 2.3 CT scanner

All simulations and measurements were performed using the 256‐slice MDCT Revolution Apex system (GE Healthcare, Waukesha, WI, USA). This scanner utilizes a TCM system known as “SmartmA”,^21^ which automatically adjusts the tube current in both the angular and longitudinal directions based on the attenuation profile obtained from the localizer radiograph. The system allows users to set a Noise Index (NI),^21^ which defines the desired noise level in the resulting images. Additionally, users can configure the minimum and maximum tube current values to optimize radiation dose while maintaining image quality during the scan. In this study, a combination of angular and longitudinal TCM was applied.

### 2.4 MC simulations and the source model

#### 2.4.1 MC simulation code

MC simulations were conducted using PHITS version 3.341.^20^ All simulations were operated in “photon transport mode”—the production and tracking of secondary electrons were excluded, with an energy cutoff set at 5 keV. In this mode, the secondary electron is assumed to be deposited locally at the photon interaction site, based on the concept of charged particle equilibrium. This assumption ensures that the photon‐only transport mode provides accurate dose estimates without explicitly simulating electron transport, while also reducing computational time.^22^ Photon interactions were simulated using cross‐section data from the EGS5 and EPDL97 libraries incorporated in PHITS, covering photoelectric effect, Compton and Rayleigh scattering, and pair production across a broad energy range. The simulations were performed on a desktop computer equipped with 32 GB of RAM, CPU (Intel®Core™ i7‐14700 at 2.10 GHz).

#### 2.4.2 Source model

**FIGURE 3:**
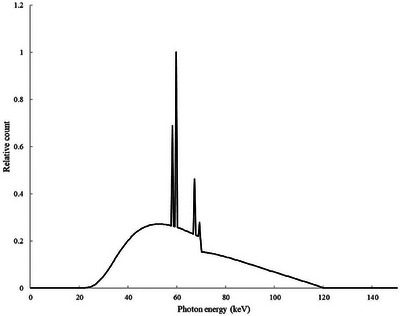
X‐ray spectrum for a tungsten target at 120 kVp with the relative intensity in a 0.5 keV interval generated using x‐ray‐spectrum‐2 software.

The CT scanner geometry was constructed using PHITS‐defined surfaces and cells, with each component modeled according to the PHITS input format. The x‐ray spectrum was generated using the x‐ray‐spectrum‐2 software,^23^ based on the semi‐empirical model developed by Tucker et al.^24^ The spectrum (Figure 3) was created using a tungsten target with a tube voltage of 120 kVp, and an energy bin width of 0.5 keV. A target angle of 10° was selected based on design parameters reported in the manufacturer's specifications,^25^ and a first half‐value layer (HVL) of 7.56 mmAl. The HVL was measured using a pencil ionization chamber (10 × 6‐3CT; Radcal Corporation) with an active volume of 3 cm^3^ connected to an electrometer (9096; Radcal Corporation). A sheet of aluminum with 99.5% purity was used as the attenuator for the x‐ray beam, and the measurement was carried out with a non‐rotating x‐ray tube setup. The bowtie filter profile was estimated using the fixed‐tube method^26^ to account for beam‐shaping effects. In this method, the x‐ray tube remained stationary at the 3 o'clock position on the gantry. The pencil ionization chamber was initially positioned at the lowest vertical point of the gantry and then raised in 10 mm increments along the y‐axis to measure exposure attenuation through the upper portion of the bowtie filter. The source‐to‐isocenter distance (62.6 cm), focal spot‐to‐detector distance (110 cm), and fan angle (47°) were determined according to the manufacturer's technical specifications.^25^ The material composition of the CT geometry was defined as follows: the bowtie filter was assumed to be aluminum (*ρ *= 2.7 g/cm^3^)^18^ and the collimator was modeled as a tungsten‐based heavy alloy (*ρ* = 17 g/cm^3^).^27^

#### 2.4.3 MC simulation condition

To calculate CT dose employing MC simulation, one full tube rotation was divided into 40 projections with 9‐degree intervals, in accordance with our previous study using the same scanner model.^18^ The T‐deposit tally was employed to calculate the energy deposition in the designated regions, representing the total absorbed energy at each location. The number of photons simulated ranged from 1 × 10^8^ to 1 × 10^9^ photons/projection to keep the statistical uncertainties below 1%, and the results from all projections were summed by using the icntl = 13 setting. Additionally, for helical scan mode, the number of projections was estimated by calculating the tube rotation from the beginning to the end of the scan, as applied in our simulation setup, in order to align with the measurement conditions.

### 2.5 Normalization factor

The absorbed dose output from PHITS is expressed in Gray (Gy)/source photon, indicating the dose delivered by individual photon. To enable a precise comparison with experimental measurements, a normalization factor (NF) is necessary to convert the simulated dose into units of Gy. The CT dose index in air (CTDI_air_) was measured at the center of the CT bore using an ionization chamber (10 × 6‐3CT; Radcal Corporation) with active volume 3 cm^3^ connected to an electrometer (9096; Radcal Corporation) and also estimated through simulation under the same conditions, which included a tube voltage of 120 kVp, tube current of 100 mA, rotation time of 1 s/rot, and an 80 mm beam width. The *NF* was calculated using the following equation:
(2)

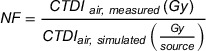

where $CTDI_{air,mesured}$ is measured CTDI in air, and $CTDI_{air,simulated}$ is simulated CTDI in air.

### 2.6 CTDI validation between measurement and simulation

The CTDI 100 (*CTDI*_100_) was performed using a 32‐cm diameter cylindrical phantom made of PMMA. A pencil ionization chamber (10 × 6‐3CT; Radcal Corporation) connected to an electrometer (9096; Radcal Corporation) was placed at the center of the phantom (*CTDI*_100_,*_c_*) and at four peripheral positions (*CTDI*_100_,*_p_*). Additionally, each location was measured three times to enhance reliability and reduce random error, and the resulting CTDI values were averaged. The weighted CTDI (*CTDI*_w_) was then calculated using the following equation:

(3) $$CTDIw\;=\frac{1}{3}\;CTDI_{100,c}+\frac{2}{3}CTDI_{100,p}$$

where $CTDI_{w}$ is the weighted CTDI, $CTDI_{100,c}$ is the *CTDI_100_* measured at the center, and $CTDI_{100,p}$ is the average of *CTDI_100_* measured at the four peripheral points.

**FIGURE 4:**
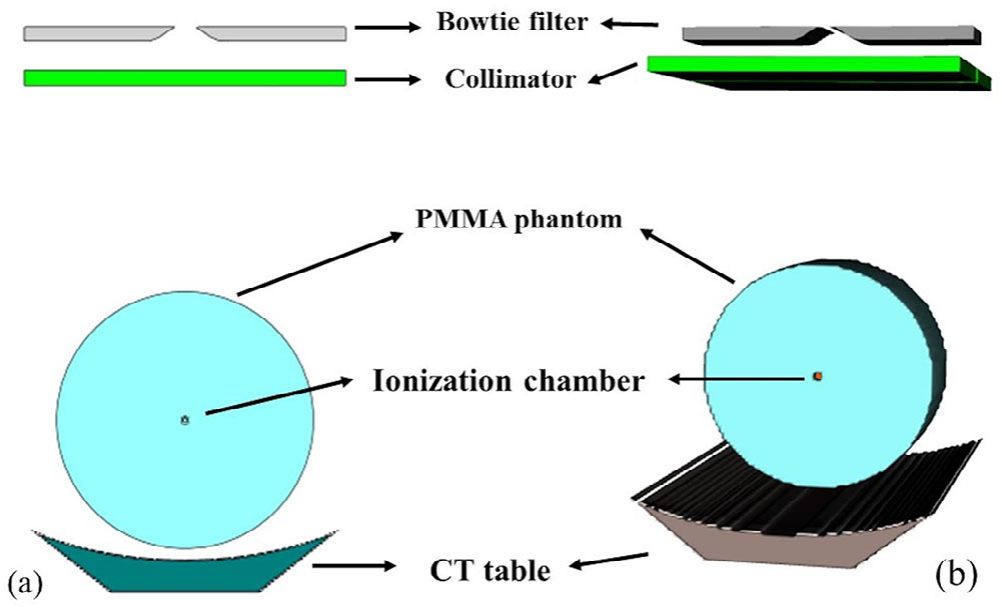
Geometry of the CT model used for CTDI_100_ simulation: (a) 2D view and (b) 3D view.

In the simulation, the exposure settings were aligned with those used in the measurements: a tube voltage of 120 kVp, tube current of 100 mA, rotation time of 1 s/rot, and an 80 mm beam width, all in non‐helical scanning mode. According to the CT model geometry described in Section 2.4.2, a PMMA phantom (*ρ* = 1.19 g/cm^3^) and a tabletop—modeled with an outer carbon fiber layer (*ρ* = 1.6 g/cm^3^) and an inner Styrofoam core (*ρ* = 0.015 g/cm^3^)^18^ ^—^were incorporated into the simulation (Figure 4). The details of the parameters were provided in the Section. 2.4.3. The CTDI values from the simulation and measurement were compared using the percentage relative difference (RD) calculated using the following equation:

(4) $$RD\left(\%\right)=\frac{simulated\;doses-measured\;doses\;}{measured\;doses}\;x100$$

### 2.7 Longitudinal TCM profile estimation

**FIGURE 5:**
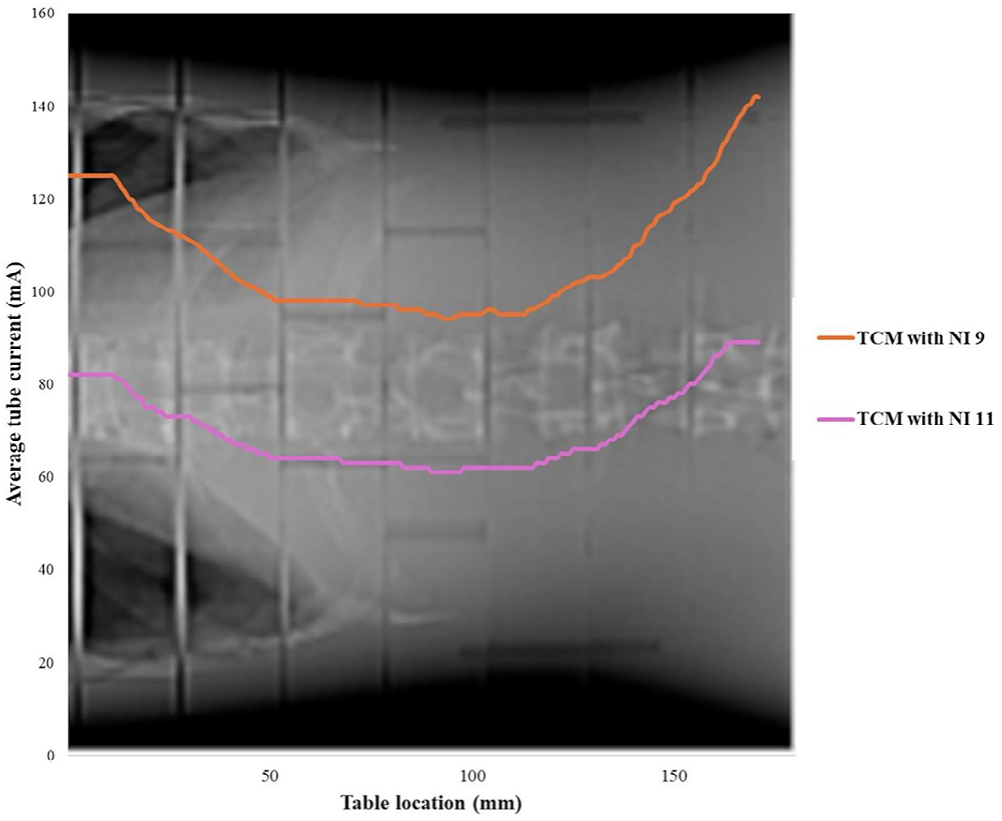
TCM profile estimation for TCM with NI 9 and NI 11.

The TCM profile was generated by plotting the average tube current per rotation against the table position (Figure 5). This method estimates the combined influence of both angular and longitudinal TCM. The tube current values were extracted from the DICOM header of post‐reconstruction CT images with a slice thickness of 0.625 mm to acquire more detailed TCM information, using R^28^ version 4.4. The effect of TCM was simulated by applying projection‐specific weighting factors, derived from the TCM profile, to the tallied results, corresponding to the tube current of each projection, while maintaining a constant total number of source particles throughout the simulation. However, the overranging image data were not accessible from the DICOM header, so the effect of the overranging region was not included in the simulation.

### 2.8 Organ dose measurements and simulations

A total of 90 RPLDs were employed, arranged in three sets of 30. Each RPLD was calibrated and inserted in a custom holder, then placed in anatomically relevant locations within the phantom to estimate absorbed doses in six abdominal organs. The number of RPLDs used per organ ranged from 2 to 10, as detailed in Table 1.

**TABLE 1:** Number of RPLDs in various organs for measurement.

Organ	Phantom slice number	Number of RPLDs
Liver	20–24	10
Stomach	20–22	7
Spleen	23–24	4
Gallbladder	23	2
Pancreas	24	3
Kidneys	25–26	4
Total		30

**FIGURE 6:**
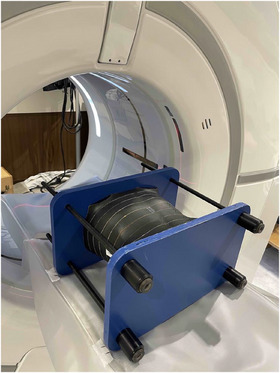
Setup of abdominal anthropomorphic phantom aligned with the laser system on the CT scanner table for organ dose measurements.

The female abdominal phantom was scanned in a supine head‐first position, from the dome of the diaphragm down to the iliac crests. The phantom was aligned using the CT scanner's laser positioning system, and reference markers were applied to ensure consistent positioning across scans (Figure 6). To reduce random errors, the organ absorbed dose at each location was measured three times. The scanning parameters for the three protocols, with and without TCM, are summarized in Table 2. The average organ absorbed dose was calculated using the following equation:

(5) $$D_{organ}=\frac{1}{n}\;\sum_{i=1}^{n}\left(M_{i}-M_{b}\right)\cdot C\;\cdot \;\frac{{\left(\frac{\mu_{en}}{\rho }\right)}_{tissue}}{{\left(\frac{\mu_{en}}{\rho }\right)}_{air}}$$

where *D*_organ_ is the average absorbed dose in each organ, *M_i_* is the measured dose from each RPLD, *M_b_* is the background dose, *C* is the calibration factor, and ($\frac{\mu_{en}}{\rho }$)_tissue_ and ($\frac{\mu_{en}}{\rho }$)_air_ are the mass energy absorption coefficients for tissue and air (cm^2^/g) for the six organs, respectively. These coefficients were obtained from the National Institute of Standards and Technology (NIST),^29^ based on the effective energy of the primary beam, which was determined from the HVL of the 120 kV spectrum. The variable n represents the total number of RPLDs inserted in each organ.

**TABLE 2:** Parameter settings for abdominal examination using the anthropomorphic phantom.

Parameters	Fixed tube current	TCM with NI 9	TCM with NI 11
Tube voltage	120	120	120
Noise index (NI)	N/A	9	11
Average tube current (mA)	300	109	70
Rotation time (s)	0.5	0.5	0.5
Pitch factor	0.508	0.508	0.508
Beam width (mm)	80	80	80
Scan length (mm)	175	175	175
CTDI_vol_ (mGy)	19.99	8.13	5.20
DLP (mGy·cm)	487.05	198.58	127.23

* N/A, not applicable.

**FIGURE 7:**
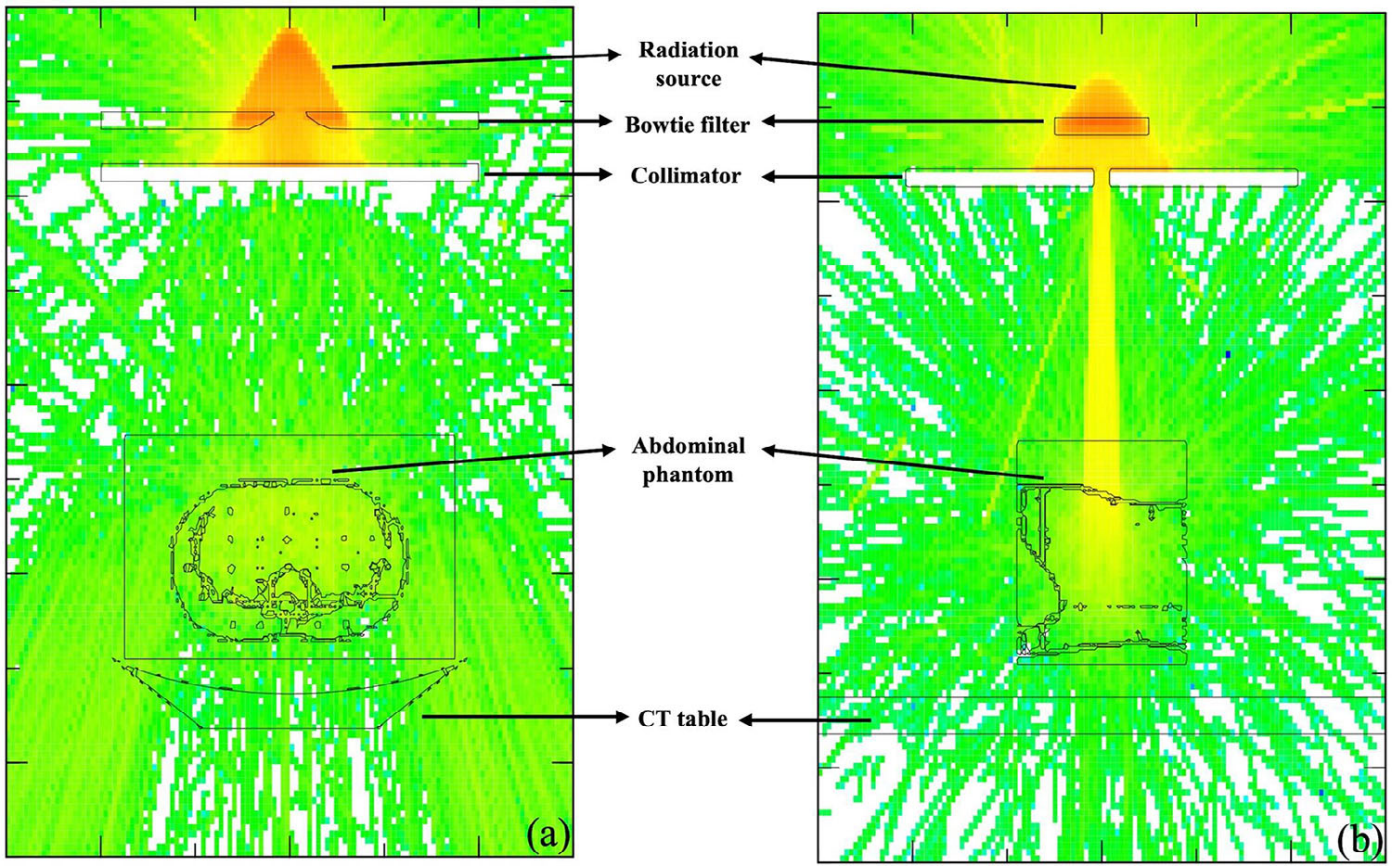
Geometry of the CT model for organ absorbed dose simulation: (a) frontal view and (b) lateral view.

For the simulation, the voxel phantom geometry was constructed from CT images and converted into the PHITS input format with a slice thickness of 5 mm using RT‐PHITS version 1.01 and the CT2PHITS module.^30^ The original CT images had voxel dimensions of 0.68 × 0.68 × 5 mm. To reduce computation time, a coarse‐graining process was performed by averaging voxel data in 4 × 4 × 1 blocks, producing a voxel size of 2.72 × 2.72 × 5 mm for the simulation. CT numbers were converted to corresponding constituent materials according to their Hounsfield unit, using a calibration approach derived from the correlation reported by Schneider et al.^31^ The associated elemental compositions and material densities were then incorporated into the subsequent simulations. The voxelized phantom was incorporated into the modeled CT scanner geometry, with the CT table included, replicating the setup used in the CTDI validation study (Section 2.6). Because the tube angle was not specified in the DICOM header of the image data, all simulations assumed a starting tube angle of 0° (12 o'clock) as shown in Figure 7. The detail of the parameters was described in the Section 2.4.3, with a total of 173 projections. The organ absorbed dose results from simulation and measurement were compared using RD (%), and dose distributions were evaluated across the three scanning protocols.

## 3 RESULTS

### 3.1 Correction factors of RPLDs

The correction factors for all 90 RPLDs ranged from 0.30 to 0.35, with an overall average of 0.32 ± 0.01. The coefficient of variation was 3.63%, indicating consistent dosimeter response across all sets. Detailed individual calibration factors are provided in Appendix A1.

### 3.2 CTDI_100_ validation between simulation and measurement

Table 3 presents the %RD between the simulation and measurement for *CTDI*_100_ at various positions, which ranged from 2% to 7%. The RD at the 6 o'clock position was higher compared to the other locations. The %RD for *CTDI*_w_ was calculated to be 3.2%.

**TABLE 3:** Relative differences between simulated and measured CTDI.

	Location	Simulation^*^ (mGy)	Measurement (mGy)	RD(%)
*CTDI_100_* at the center	Center	3.67	3.59 ± 0.002	2.07
*CTDI_100_* at the periphery	12 o'clock	7.92	7.75 ± 000	2.23
	3 o'clock	7.86	7.70 ± 0.005	2.13
	6 o'clock	7.41	6.87 ± 0.003	7.87
	9 o'clock	7.88	7.71 ± 0.002	2.19
*CTDI*_w_		6.40	6.20	3.21

* All simulated doses have statistical uncertainties less than 0.20%.

Measurement data are presented as Mean ± SD.

### 3.3 Comparison of organ dose between simulations and measurements

The %RDs, calculated using the measured values as the reference, between the average simulated and measured organ absorbed doses for the liver, gallbladder, stomach, spleen, pancreas, and kidneys were within the ranges of –7.00% to 5.89%, −14.0% to 6.86%, and −9.23% to 7.87% for fixed tube current, NI 9, and NI 11, respectively. However, the kidneys exhibited a slightly larger discrepancy, reaching up to −14% difference for NI 9, as detailed in Table 4. When scanning was performed using the TCM mode, the average organ absorbed doses in both measurements and simulations decreased by up to 66.6% for NI 9 and 78.6% for NI 11, compared to the fixed tube current, as shown in Table 5.

**TABLE 4:** Comparison of organ absorbed doses and RDs between simulation and measurement.

	Fixed tube current		RD (%)	TCM with NI 9		RD (%)	TCM with NI 11		RD (%)
Organ	Simulation^a^ (mGy)	Measurement (mGy)		Simulation^a^ (mGy)	Measurement (mGy)		Simulation^a^ (mGy)	Measurement (mGy)	
Liver	32.4	33.3 ± 0.43	−2.87	11.5	11.6 ± 0.20	−0.85	7.41	7.48 ± 0.14	−0.96
Stomach	32.1	32.5 ± 0.77	−1.09	11.8	11.4 ± 0.36	3.38	7.50	7.31 ± 0.16	2.56
Gallbladder	37.4	35.3 ± 0.36	5.89	12.5	11.7 ± 0.08	6.86	8.01	7.43 ± 0.33	7.87
Spleen	32.6	34.2 ± 0.42	−4.46	11.1	12.0 ± 0.44	−7.97	7.17	7.59 ± 0.25	−5.49
Pancreas	35.1	36.4 ± 0.89	−3.61	11.9	12.4 ± 0.27	−3.59	7.79	7.78 ± 0.02	0.07
Kidneys	30.1	32.3 ± 0.18	−7.00	10.9	12.8 ± 0.09	−14.0	7.24	7.97 ± 0.04	−9.23

^a^ All simulated doses have statistical uncertainties less than 0.25%.

Measurement data are presented as Mean ± SD.

**TABLE 5:** Percentage dose reduction in both measurement and simulation for the TCM mode.

	%Dose reduction in measurement		%Dose reduction in simulation	
Organ	TCM with NI 9	TCM with NI 11	TCM with NI 9	TCM with NI 11
Liver	65.2	77.5	64.5	77.1
Stomach	64.9	77.5	63.2	76.6
Gallbladder	65.7	79.0	66.6	78.6
Spleen	65.8	77.8	66.0	78.0
Pancreas	65.9	78.6	66.1	77.8
Kidneys	60.4	75.3	63.8	75.9

### 3.4 Comparison of dose distributions across the three protocols

**FIGURE 8:**
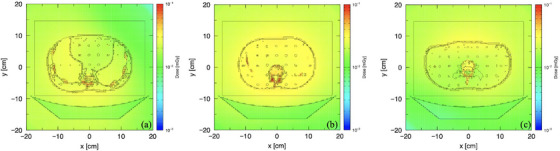
Dose distribution in the (a) upper, (b) middle, and (c) lower abdominal regions under fixed tube current.

**FIGURE 9:**
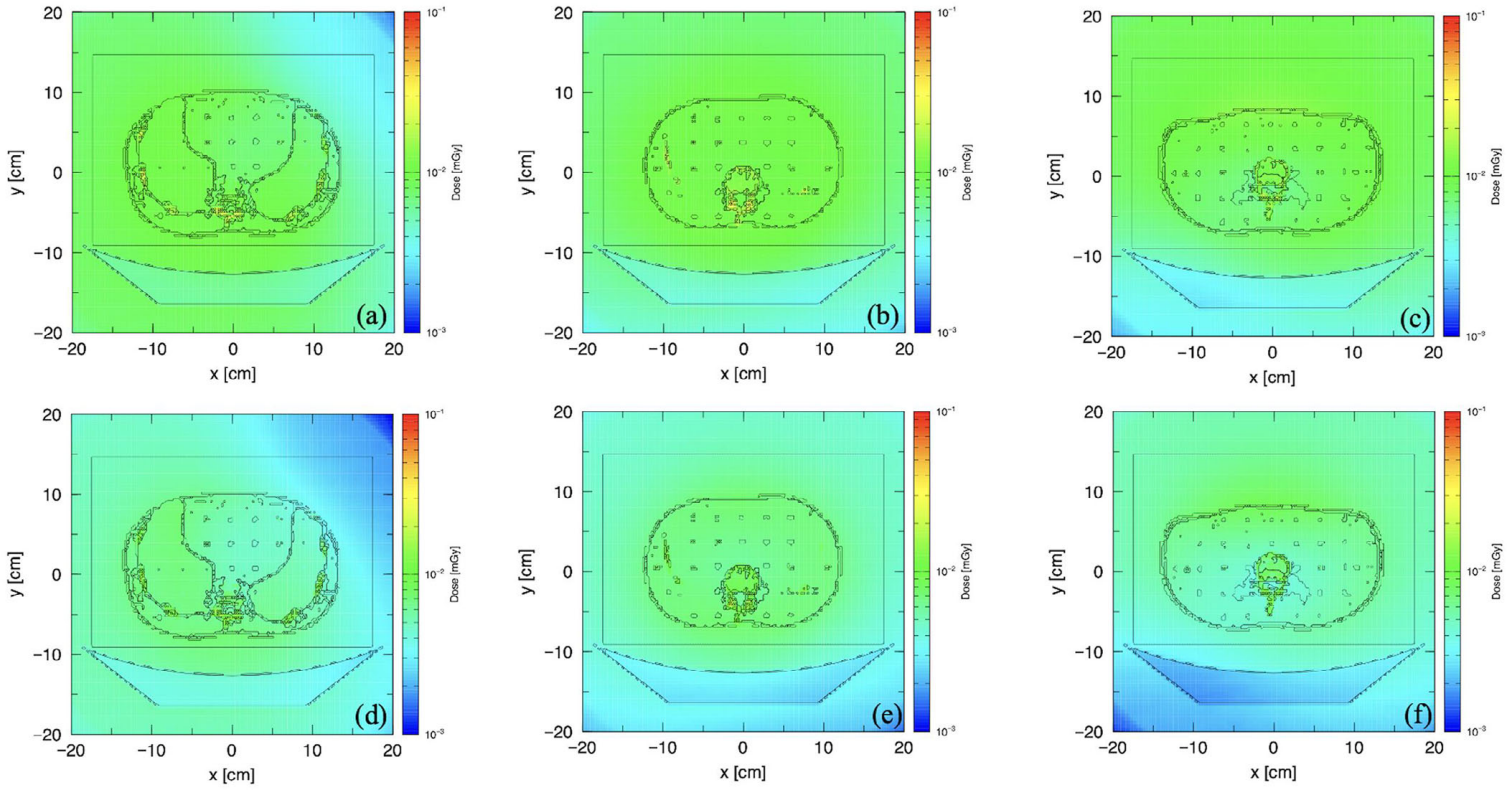
Dose distribution in the upper (a and b), middle (b and e), and lower abdominal regions (c and f) for TCM with NI 9 (a–c) and NI 11 (d–f).

The dose distribution under fixed tube current was uniform throughout all abdominal regions. However, the bone received a slightly higher dose compared to the other organs (Figure 8). Although the radiation dose varied according to the NI setting, the dose distribution pattern with TCM resembled that of the fixed tube current (Figure 9). Nevertheless, a more uniform dose distribution was seen in the lower abdominal region (Figures 9c,f).

## 4 DISCUSSION

This study estimated the absorbed doses in six organs of an adult phantom using both the fixed tube current technique and TCM. The TCM profile was derived from longitudinal TCM data extracted from the DICOM header and applied as a weighting factor in the simulations. MC simulations were validated by comparing the results with direct measurements obtained from RPLDs placed inside the phantom. Furthermore, the dose distribution was simulated to visualize the absorbed dose on CT images, illustrating the spatial distribution of radiation. These dose distributions were then compared across three different scanning protocols.

### 4.1 CTDI validation between simulations and measurements

The simulated *CTDI*_100_,*_c_* and *CTDI*_100_,*_p_* values in the body phantom were slightly overestimated relative to the measured values. This is mainly attributed to uncertainties in the simulation, such as the CT model geometry, the x‐ray spectrum, and the bowtie filter.^32^ Moreover, the %RD of *CTDI*_100_ at the 6 o'clock position was higher than at other positions, potentially due to insufficient attenuation by the CT table.^32^ Since the exact material composition of the CT table was not disclosed, it was modeled using approximated properties, as described in Section 2.6. This approximation may have contributed to the underestimated attenuation observed at that position. Accordingly, the %RD of *CTDI*_w_ showed good agreement between simulation and measurement, with a difference of 3.2%. Mendes et al.^33^ and Deak et al.^34^ reported %RD values of 2% and 5.6%, respectively, which are comparable to those in this study. These findings indicate that the PHITS simulation accurately represents dose calculations in the phantom.

### 4.2 Comparison of organ dose between simulations and measurements

The simulated absorbed doses for most organs were slightly lower than the measured values. The %RDs between simulation and measurement for organ absorbed doses, including the liver, gallbladder, stomach, spleen, and pancreas across all protocols, as well as the kidneys under fixed tube current and TCM with NI 11, were within ± 10%, indicating good agreement, consistent with findings from a previous study.^35^

This difference can be attributed to the abdominal phantom's composition is more complex and heterogeneous compared to the PMMA phantom, resulting in increased internal scatter during measurements.^36^ The gaps between phantom slices during assembly may have contributed to measurement uncertainty. Although the RPLDs were calibrated according to the applied kVp and its corresponding HVL, residual energy dependence may have influenced the measurement results. Moreover, beam hardening within the phantom may also have contributed to variations in the measured dose.^37^

Additionally, the primary sources of uncertainty in the simulation include model geometry, the x‐ray spectrum, and the bowtie filter, as previously noted. These factors can affect beam shaping and scatter estimation. The assumed material compositions and density assignments may have contributed to discrepancies between measured and simulated doses. In particular, the use of CT‐to‐material calibration curves designed for soft tissue equivalents can introduce uncertainties when applied to phantoms composed of synthetic or heterogeneous materials. This mismatch may lead to deviations in attenuation properties and dose deposition characteristics, especially in structural components of the phantom.^38^

The simulated dose for the kidney under NI 9 was found to be 14% lower than the measured value, which may be due to differences in the tube start angle between the simulation and the measurement. Long et al.^12^ and Fujii et al.^39^ reported that variations in the tube start angle have the greatest effect on organs closer to the surface, such as the thyroid, kidneys, and adrenal glands. Incorporating the actual tube start angle could improve the accuracy of dose estimation for peripheral organs and enhance the robustness of the estimated TCM approach. Moreover, the impact of the overranging region, defined as contributions from scattered radiation at the start and ends of the scan, on organs near the scan boundaries was not considered because the DICOM header did not contain tube current information for the region. The lack of this information likely contributed to the underestimation of the simulated kidney dose, due to its anatomical location near the inferior edge of the scan range.^34^

In the simulation of the TCM technique, the longitudinal TCM profiles were derived from the DICOM header. The %RDs between simulation and measurement were comparable across NI 9 and NI 11, and the fixed tube current condition. For instance, the stomach and gallbladder tended to be slightly overestimated, while other organs generally showed underestimation across all protocols. Khatonabadi et al.^40^ validated an MC model for TCM scans and found that the longitudinally approximated TCM method closely matched the estimated TCM profile extracted from raw data, with differences within 3% for lungs and breasts in all models. Fujii et al.^41^ reported that, when applying a longitudinally approximated TCM curve to an adult abdomen‐pelvis voxel phantom, the organ absorbed doses estimated by simulation with ImpactMC software differed from measured values by between 1.5% lower and 10% higher. Akhavanallaf et al.^42^ reported abdominal organ doses in a CIRS adult phantom from whole body CT examinations using the MCNPX code for simulation, incorporating a longitudinal TCM curve, and observed overestimations of 2.3% to 24% relative to measured values. Stepusin et al.^16^ similarly utilized DICOM‐based tube current information to extract the longitudinal dose profiles and found that their simulations underestimated measured values by up to 12%. These simulation outcomes were comparable to results obtained using both angular and longitudinal TCM curves. Therefore, the longitudinal TCM dose profiles in this study can be regarded as accurate and consistent with the measured data.

Additionally, Long et al.^12^ estimated organ absorbed doses using both measurements and MC simulations with MCNPX, reporting differences between simulation and measured values ranging from 9.8% lower to 13.4% higher in an adult phantom. Deak et al.^34^ estimated organ absorbed dose using a liver phantom under the TCM technique and found that the simulation results were within 10% of the measured values. The findings of our study align with these previous reports and demonstrate the ability to accurately estimate organ absorbed doses.

Finally, the use of the TCM technique has a significant impact on radiation dose. The NI is a parameter that controls the noise level in images and is inversely related to radiation dose; as NI increases, the radiation dose decreases,^43^ as seen in the comparison between TCM with NI 9 and NI 11. However, the clinical application of TCM requires careful optimization to balance radiation dose reduction while preserving diagnostic image quality.

### 4.3 Comparison of the dose distribution across all protocols

The color scale on the dose distribution map not only offers a visual representation of radiation dose across the area but also supports both qualitative and quantitative analyses of its complexity.^34^ In this study, higher dose values were observed in the costal and vertebral regions compared to the surrounding organs, which is due to the bone's greater x‐ray absorption compared to soft tissue.^44^ Because of the tissue composition, the abdomen shows less variation in attenuation than other regions like the chest or pelvis. This leads to smaller adjustments in tube current along the phantom,^45^ resulting in a dose distribution pattern under TCM that resembles that of the fixed tube current. However, a more uniform dose distribution appeared in the lower region of the phantom with TCM at NI 9 and NI 11, attributed to the combined effects of both longitudinal and angular modulation adjusting the tube current in both scanning directions^46^

### 4.4 Sources of error

As discussed above, the primary sources of error include (1) anatomical discrepancies and unknown material composition of the physical phantom, and (2) uncertainties in source modeling related to scanner geometry and scan acquisition parameters (e.g., tube start angle and overranging). Additionally, there are several other possible factors that may have influenced both the measurements and simulations in this study. On the measurements side, the RPLD system used is reported by the manufacturer to exhibit reproducibility within ± 2% at 1 mGy and linearity within ± 5% across the clinical dose range. Although laser alignment and reference markings were applied to ensure consistent phantom placement, minor variations in setup may still have contributed to measurement uncertainty. On the simulation side, source model uncertainties are associated with the x‐ray implementation in PHITS, which was based on measurements obtained using the Radcal ionization chamber (Section 2.4.2). This chamber carries a calibration uncertainty of ± 7% and an energy dependence of ± 5%. MC simulations were performed with a large number of particle histories to ensure that statistical uncertainty in organ dose estimates remained below 1%. The statistical uncertainty of CTDI simulations and organ dose estimations was less than 0.20% and 0.25%, respectively, and is therefore considered negligible compared to measurement‐related uncertainties.

### 4.5 Advantages and applications of the proposed method

This method provides a practical and efficient alternative for estimating organ dose in CT examinations using TCM. It is applicable not only to phantom studies but also to patient‐specific evaluations, as it requires no additional information beyond routine CT images, making it particularly suitable for retrospective analyses. By extracting tube current profiles directly from the DICOM header, the approach enables individualized dose estimation in a straightforward and noninvasive manner. Its compatibility with both patient data and MC simulation tools supports the generation of quantitative dose distributions, which are crucial for assessing radiation‐related risks, especially in radiosensitive organs. Moreover, this approach contributes to dose optimization protocols and minimizes radiation dose. Overall, these findings help address current challenges in CT dosimetry and provide meaningful insights for both clinical practice and research applications.

### 4.6 Limitations

This study has several limitations, including the use of only one CT scanner and one type of phantom. Although the phantom represents an average human size, organ absorbed dose estimates may differ for patients with varying body types, such as those who are underweight or obese. Nevertheless, this method could be adapted to calculate organ absorbed doses from patient‐specific data. Furthermore, the TCM dose profile validation relied solely on physical measurements; estimating dose distributions for systems from other manufacturers would require different modeling approaches.

## 5 CONCLUSION

The CTDI values obtained from the MC simulations closely matched the direct measurements, confirming the accuracy of PHITS for dose estimation. Most differences in organ absorbed doses were within ± 10%, though slightly larger discrepancies were observed for the kidneys under the TCM setting with NI 9. The longitudinal tube current values extracted from the DICOM header were successfully used as TCM profiles in the simulations. The TCM technique was shown to reduce organ doses and provide a more uniform dose distribution in the lower abdomen compared to the fixed tube current method. These findings validate the proposed method as an effective approach for estimating organ absorbed doses in abdominal CT scans using both fixed and modulated tube current techniques.

## AUTHOR CONTRIBUTIONS

*Preparation of manuscript and designing the experiments*: Chatnapa Nuntue. *Collection and analysis of data*: Chatnapa Nuntue, Kosuke Matsubara, Shu Watanabe, Kotaro Fukushima, and Khajonsak Tantiwetchayanon. *Supervision of work; providing critical feedback; discussing the results; and reviewing the manuscript*: Kosuke Matsubara

All authors contributed to the drafting of the manuscript and approved the final version.

## CONFLICT OF INTEREST STATEMENT

The authors declare that they have no known competing financial interests or personal relationships that could have appeared to influence the work reported in this paper.

## Data Availability

The data cannot be made publicly available upon publication because no suitable repository exists to host them. The data supporting the findings of this study are available upon reasonable request from the authors.

## APPENDIX A

### A.1

**TABLE A1:** The correction factors *(C)* for 90 RPLDs irradiated under identical CT conditions, calibrated against an ionization chamber.

RPLD No.	*C*	RPLD No.	*C*	RPLD No.	*C*
1	0.30	31	0.32	61	0.33
2	0.31	32	0.30	62	0.34
3	0.30	33	0.30	63	0.32
4	0.31	34	0.34	64	0.31
5	0.34	35	0.32	65	0.33
6	0.31	36	0.33	66	0.31
7	0.32	37	0.32	67	0.31
8	0.31	38	0.32	68	0.31
9	0.32	39	0.34	69	0.31
10	0.31	40	0.34	70	0.32
11	0.33	41	0.32	71	0.32
12	0.32	42	0.30	72	0.31
13	0.32	43	0.34	73	0.31
14	0.33	44	0.31	74	0.32
15	0.34	45	0.30	75	0.32
16	0.33	46	0.30	76	0.31
17	0.33	47	0.32	77	0.33
18	0.32	48	0.32	78	0.34
19	0.31	49	0.32	79	0.33
20	0.32	50	0.32	80	0.33
21	0.31	51	0.32	81	0.35
22	0.32	52	0.31	82	0.31
23	0.33	53	0.31	83	0.32
24	0.31	54	0.32	84	0.33
25	0.30	55	0.32	85	0.32
26	0.31	56	0.33	86	0.32
27	0.31	57	0.30	87	0.31
28	0.31	58	0.34	88	0.30
29	0.32	59	0.32	89	0.33
30	0.32	60	0.35	90	0.32
